# Authors’ reply to the comment from Benavides-Zora et al.

**DOI:** 10.1186/s13054-023-04547-x

**Published:** 2023-06-29

**Authors:** Yuki Kotani, Alessandro Pruna, Todd C. Lee, Dominik Roth, Giovanni Landoni

**Affiliations:** 1 Department of Anesthesia and Intensive Care, IRCCS San Raffaele Scientific Institute, Via Olgettina 60, 20132 Milan, Italy; 2 School of Medicine, Vita-Salute San Raffaele University, Via Olgettina 58, 20132 Milan, Italy; 3 Department of Intensive Care Medicine, Kameda Medical Center, 929 Higashi-cho, Kamogawa, Chiba 296-8602 Japan; 4 Division of Infectious Diseases, Department of Medicine, McGill University, Montreal, QC Canada; 5 Department of Emergency Medicine, Medical University of Vienna, Währinger Gürtel 18-20, 1090 Vienna, Austria

With the goal of including all available randomized controlled trials (RCTs) on propofol, we meta-analyzed 252 trials reporting mortality at 18 different time-points and pooled data at the longest follow-up available as previously validated [1]. Even if our analysis would violate the proportional hazards assumption, this would only lead to an underestimation of pooled effect size, further strengthening the robustness of our findings [2]. Sensitivity analyses on the five most frequently reported time-points arrived at a similar magnitude and direction as the primary analysis. Interestingly, further mRCT evidence (not included in our meta-analysis because of inclusion criteria) confirms a detrimental effect of propofol on survival persisting up to one year [3].

We adopted an intention-to-treat approach when extracting mortality data to prevent exaggeration of treatment effects that can occur in per-protocol analyses [4]. In the cardiovascular setting, including Likhvantsev et al. study using the evaluable patients’ data (not the correctly extracted intention-to-treat data), the impact remains statistically significant (RR, 1.36; 95% CI, 1.06–1.76; Additional file 1: Table S1).

We acknowledge clinical heterogeneity across different subgroups. However, our subgroup analyses consistently showed results similar to our main analysis in magnitude and direction. The debate “fixed versus random-effects models” goes beyond the scope of this letter and was addressed in another reply. However, when repeating the analysis using random-effects model and trim-and-fill approach, results remained consistent with the main analysis (Additional file 1: Table 1).

The composition of intensive care unit (ICU) and perioperative RCTs in our analysis was similar to Roth et al. in which 16% of included studies were set in medical ICUs [1]. Although our meta-analysis also included perioperative studies, more than 50% of the deaths occurred in ICU studies.

The ICU subgroup also had > 10% relative mortality increase (15% vs. 13%) with Bayesian approach indicating 75.7% probability of harm. Since the outcome is death, it is maybe cavalier to dismiss such probability as “no difference,” especially given a pediatric RCT suggested harm leading to a FDA warning [5] and the manufacturer’s promise for a second RCT which was never conducted. With millions of patients exposed, and the potential for increased mortality, we would disagree with the suggestion this is “spin.”

Considering the availability of other sedation strategies (e.g., alternative hypnotic agents, sedation rotation, dose minimization), we believe our findings warrant careful consideration. Our study aims to raise awareness about potential propofol-associated risks and support the kind of pragmatic mRCTs that clinicians need and patients deserve.

## Supplementary Information

**Additional file 1**. Supplemental Table 1.

## Data Availability

Further information on the original manuscript is available from the corresponding authors upon reasonable request.

## Abbreviations

CI: Confidence interval
FDA: Food and Drug Administration
ICU: Intensive care unit
mRCT: Multicenter randomized controlled trials
RCT: Randomized controlled trial
RR: Risk ratio
