# Supplementary material for: Authors’ reply to the comment from Benavides-Zora et al
Source: Crit Care. 2023 Jun 29;27:255. doi: 10.1186/s13054-023-04547-x (PMC10311869; doi:10.1186/s13054-023-04547-x)
Supplement: Supplementary file 1 — Additional file 1. Supplemental Table 1. [file 13054_2023_4547_MOESM1_ESM.docx]

Supplemental Table 1. Exploratory analyses for the overall population with different mortality time points and cardiovascular surgery subgroup with different data extraction strategy for Likhvantsev et al study

| Data extraction strategy | Model | Method | Risk ratio  (95% CI) | Probability of  risk ratio >1.0 |
| --- | --- | --- | --- | --- |
| Overall population |  |  |  |  |
| Mortality at the longest follow-up available | Fixed-effects | M-H | 1.10 (1.01-1.20) | 98.4% |
| Mortality at the longest follow-up available | Random-effects | M-H | 1.05 (0.98-1.13) | 91.0% |
| Mortality at the longest follow-up available, with trim and fill^*^ | Random-effects | M-H | 1.08 (1.004-1.15) | 98.7% |
| Mortality at the time point closest to 30 days | Fixed-effects | M-H | 1.06 (0.97-1.16) | 89.9% |
| Cardiovascular surgery subgroup |  |  |  |  |
| Intention to treat | Fixed-effects | M-H | 1.46 (1.13-1.89) | 99.8% |
| Evaluable population | Fixed-effects | M-H | 1.36 (1.06-1.76) | 99.1% |

Abbreviations: M-H, Mantel-Haenszel; CI, confidence interval

^*^ 24 studies were added.
